# Special Issue “Mineral Composite Materials Produced with Waste/Recycled Components”—Editorial Note and Critical Review of the Problems

**DOI:** 10.3390/ma16113911

**Published:** 2023-05-23

**Authors:** Agata Stempkowska, Tomasz Gawenda

**Affiliations:** Department of Environmental Engineering, Faculty of Civil Engineering and Resource Management, AGH University of Science and Technology, Mickiewicza 30 Av., 30-059 Cracow, Poland; gawenda@agh.edu.pl

## 1. Introduction

Modern materials science encompasses a range of interdisciplinary issues and goes beyond the conventional curricula of universities and technical courses. Today, a holistic approach is important, so waste materials should be treated as raw materials. Of particular interest seems to be the use of mineral and organic waste in advanced composites. A wide range of materials can be used in recycling; these materials can be artificially obtained aggregates, simple and polymeric concretes, clay-cement binders, granules that work as sorbents, filters or soil substrates, and many others. A common feature is the treatment of waste/recycled raw materials as a full component of manufactured composites. The word “waste” connotes a material devoid of value and utility. However, developing technology makes it possible to use waste materials to produce a range of high-performance composite products that can also be recycled. There is a pressing need for research and development to maximize the benefits of using recycled waste materials in composite products.

## 2. Impact Assessment

**Figure 1:**
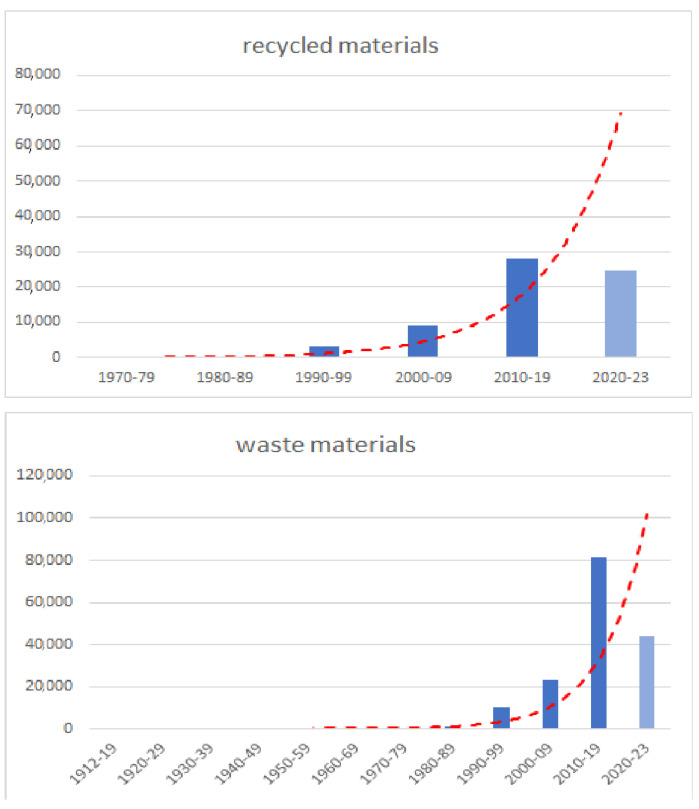
Number of available publications in the field of recycled materials and waste materials.

An assessment of the impact of a particular topic can be demonstrated by the number of scientific publications on that topic. A good example of this approach is bibliometric analysis, by means of which the qualitative and quantitative analysis of scientific publications registered in databases can be carried out and interesting results can be obtained. In general, a relatively large number of publications on the use of alternative materials in the manufacture of useful materials can be observed, but in order to verify this, a detailed analysis was carried out for records registered in the Web of Science (WoS) database. The WoS Core Collection database was selected, and waste materials and recycled materials were set as the primary search criteria. The all-fields option was selected in the documents searched, and the period analyzed was all available years up to the first quarter of 2023 (Figure 1). The search was conducted from 12 to 14 April, 2023. Search results of 177,931 records for waste materials and 70,547 records for recycled materials were obtained. It should be noted that publications on recycling topics began to appear only in 1970, while the first articles on waste materials were registered in 1912. The number of publications registered in the WoS database by year is shown in Figure 1.

In the overall trend, it is important to note the exponential increase in the number of publications starting in the 1990s. The results in this regard indicate that the issues of the use of waste and recycling raw materials are of great interest; an interest that has been growing, especially in the last decade.

The next step was to refine the search, and publications from 2010 onward were provided for further analysis in an effort to take into account recent research developments. The records obtained (143,312 for waste materials and 39,766 for recycled materials) were analyzed under the following categories: region/country, publication source (journal name), research area, and scientific institution. Table 1 shows the most common research areas of the searches. The results of the two searches are similar, mainly in the environmental and material sciences category but also in the chemical sciences group. The three most popular areas are environmental sciences, material science multidisciplinarity, and engineering environmental. It is worth mentioning that one record (publication) can be assigned to several areas, so the cumulative percentage in Table 2 is higher than 100%. This can be clearly seen in the first four categories as the cumulative percentage share for them alone exceeds 100 percent; many publications on problems with waste and recycling materials must be associated with at least two of the leading areas. It should be noted that multi-author publications are often found in several research areas.

**Figure 2:**
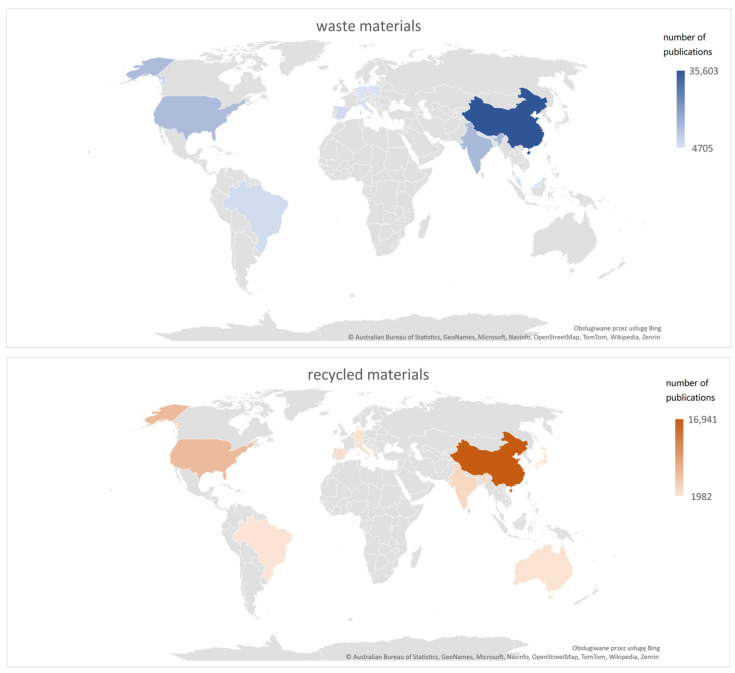
Top 10 countries with the highest number of publications in Web of Science database.

Figure 2 shows that the countries that generate the most publications on waste and recycled materials are China, USA, and India, with China producing ¼ of the world’s research and articles in this field.

Table 2 shows the titles of the journals in which the most frequently published articles in the field in question are published. While the position of individual journals differs, the same journals appear on both lists. It is worth noting that the frequency of publication does not depend on the IF of the journal.

An analysis of the obtained records from the WoS database grouped by publisher is presented in Table 3. It contains the 10 publishers with the highest number of publications on waste and recycled materials. The first three publishers that publish the most on this research topic are, in both cases, Elsevier, Springer Nature, and MDPI. Note that many of the journals are owned by the same publisher. For example, in 2010, in the journal *Materials*, the topics published by MDPI accounted for 0.83% of published materials, while in 2023, the number was 10.73%.

When it concerns research on waste materials, Chinese researchers from the Chinese Academy of Sciences are the most active, followed by those from Egypt and India. The recycling phenomenon is also most extensively researched in China, with as many as 5 research units in the top 10 (Table 4).

## 3. Qualitative Analysis and Discussion

The qualitative analysis was narrowed down to the two most common research areas, which are materials science multidisciplinarity and environmental science.

### 3.1. Scientific Area

By analyzing the content, we draw the conclusion that the dominant issue emerging from the topics of waste and recycled materials is the technology of building materials and, in particular, concrete technology (Table 5). It can be concluded that these issues are present in virtually every scientific and economic field; from mineral and biotic resources, through the broader chemical and physical sciences to geotechnics. In the areas analyzed, a very important component is the study of water pollution and treatment, land contamination, fuel and energy use of natural resources, and sustainable development. It can be concluded that waste is treated as a reusable raw material after appropriate treatment and processing. This demonstrates humans’ conscious and holistic approach to nature and its resources.

### 3.2. Content Analysis and Discussion

A detailed content analysis addresses mineral composites that can be produced with waste and recycled materials. However, it is impossible to list all the titles and authors who deal with waste and recycling in their works. This paper examines a selection of recent publications in this field, written between 2022 and 2023, with a focus on structural, pavement and building materials.

The use of recycled materials is an important environmental issue. Large quantities of waste raw materials recovered from the demolition of asphalt road structures indicate the need to find new ways to use them. In the case of road rehabilitation projects, large quantities of secondary raw materials are mostly recovered in the form of reclaimed asphalt pavements, reclaimed concrete, and recycled aggregate [1,2,3,4]. The amount of waste materials and by-products is increasing and threatens environmental safety. Some of this waste can be used in the production of construction materials, such as concrete, or substitute cementitious materials [5,6,7,8,9,10,11,12]. Research is also being conducted using expired cement [13]. Meeting the current demand for concrete does not only require the extraction of tons of gravel and sand, but also the burning of large amounts of fossil fuel resources in the cement-burning process. Therefore, concrete recycling is crucial in achieving a materially efficient society, especially with different categories of concrete and the goal of phasing out fossil fuels [14,15,16,17]. Often, ground glass is also used as an alternative material for concrete [18,19,20,21].

A good direction for reusing fibers from textile waste is to develop innovative and sustainable materials for use in construction. Currently, managing the large amounts of textile waste generated and reducing the damage that this waste causes to the ecosystem involves finding solutions to reuse it, for example, as alternative reinforcement for concrete [22,23].

Interesting applications of waste materials such as fly ash, iron slag and silica fume can be found in fired materials such as bricks, facade bricks, tiles [24,25,26,27]. However, the authors of the above publications point out the difficulties in using waste materials. Mineral wastes, despite homogenization processes, are characterized by low compositional stability by the presence of soluble compounds. Such compounds can cause efflorescence on the surface of plastics, which causes defects in use, especially in the production of finishing elements.

The use of waste as a construction material or soil stabilization is an emerging field in the construction industry. The introduction of new supplementary materials to strengthen local soils using industrial waste is an inexpensive and more effective method of soil improvement. High-calcium asphalt concrete production waste is being used to stabilize low-quality soil as a sub-base material for road structures. Asphalt waste dust is successfully used as a sub-base material in road structures in accordance with standards for pavement materials [28,29,30]. Soils can be easily strengthened using low-energy stabilization methods [31].

Modern and promising construction materials are alkali-activated binders from numerous industrial wastes and by-products. Glass powders, cementitious substitute materials, mineral powders, slag, and many others are used here [32,33,34,35].

## 4. Conclusions

1. An analysis of articles in the field of waste and recycled materials registered in the WoS database indicates that the environmental direction is of crucial importance, and its role in research is becoming increasingly important;
2. Environmental issues, such as the limitation of natural resources and large amounts of waste, are leading the way in developing a culture of sustainable construction. The two main environmental problems are the depletion of natural resources and the disposal of waste materials generated during various processes;
3. The problem of disposal and management of solid waste materials has become one of the main environmental, economic, and social problems. Not only does the use of solid waste in the production of construction materials solve the problem of disposal, but it also helps transform waste into useful and profitable products.

## Figures and Tables

**Table 1:** Most common research areas associated with publications on the topic.

Waste Materials			Recycled Materials		
Research Area	Number of Publications	[%]	Research Area	Number of Publications	[%]
Environmental Sciences	42,057	33.68	Material Science Multidiscyplinary	16,846	42.36
Material Science Multidiscyplinary	39,710	31.80	Environmental Sciences	10,317	25.94
Engineering Environmental	32,329	25.89	Engineering Environmental	8040	20.22
Engineering Chemical	16,780	13.44	Engineering Civil	7353	18.49
Energy Fuels	14,535	11.63	Construction Building Technology	6811	17.13
Engineering Civil	12,222	9.79	Green Sustainable Science Technology	5832	14.67
Green Sustainable Science Technology	11,919	9.54	Chemistry Physical	4689	11.79
Chemistry Multidiscyplinary	11,530	9.23	Engineering Chemical	4273	10.75
Chemistry Physical	11,185	8.96	Chemistry Multidiscyplinary	4223	10.62
Construction Building Technology	11,042	8.84	Physics Applied	3456	8.69

**Table 2:** Top 10 journals with the largest number of publications in the discussed field.

Waste Materials				Recycled Materials			
Journal Title	Number of Publications	[%]	IF	Journal Title	Number of Publications	[%]	IF
Journal of Hazardous Materials	5160	3.60	14.224	Construction and Building Materials	3119	5.44	7.693
Construction and Building Materials	4476	3.12	7.693	Journal of Cleaner Production	1821	3.18	11.072
Journal of Cleaner Production	3515	2.45	11.072	Materials	1246	2.17	3.748
Advanced Materials Research	2566	1.79	25.809	Advanced Materials Research	1069	1.87	25.809
Materials	2490	1.74	3.748	Journal of Hazardous Materials	946	1.65	14.224
Materials Today Procedings	2134	1.49	-	Waste Management	859	1.50	8.816
IOP Conference Series, Materials Science and Engineering	1987	1.39	-	Resources Conservation and Recycling	835	1.46	13.716
Waste Management	1966	1.37	8.816	Sustainability	813	1.42	3.889
Sustainability	1542	1.07	3.889	ACS Suistainable Chemistry Engineering	554	0.89	9.224
Environmental Science and Pollution Research	1384	0.96	5.190	Journal of Materials in Civil Engineering	511	0.97	3.651

**Table 3:** Top 10 publishers with the most publications on waste and recycled materials.

Waste Materials			Recycled Materials		
Publisher	Number of Publications	[%]	Publisher	Number of Publications	[%]
Elsevier	52,742	36.78	Elsevier	20,168	35.20
Springer Nature	15,640	10.91	Springer Nature	5424	9.47
MDPI	10,595	7.39	MDPI	4439	7.75
Wiley	5836	4.07	Wiley	2978	5.20
Taylor&Francis	4890	3.41	Amer Chemical Soc	2600	4.54
Amer Chemical Soc	4582	3.20	Taylor&Francis	1926	3.36
Trans Tech Publications Ltd.	4065	2.83	Royal Soc Chemistry	1825	3.18
Iop Publishing Ltd.	3572	2.49	Trans Tech Publications Ltd.	1814	3.17
Royal Soc Chemistry	3373	2.35	IOP Publishing Ltd.	1054	1.84
Sage	1580	1.10	Sage	966	1.67

**Table 4:** Top 10 best publishing research units.

Waste Materials			Recycled Materials		
University/Institute	Number of Publications	[%]	University/Institute	Number of Publications	[%]
Chinese Academy of Sciences	3409	2.37	Chinese Academy of Sciences	1728	3.01
Egyptian Knowledge Bank EKB	2443	1.70	Centre National De la Recherche Scientifique CNRS	961	1.67
Indian Institute of Technology System IIT System	2120	1.47	Egyptian Knowledge Bank EKB	727	1.27
Centre National De la Recherche Scientifique CNRS	2089	1.45	Indian Institute of Technology System IIT System	651	1.13
National Institute of Technology NIT System	1764	1.23	United States Department of Energy DOE	600	1.05
United States Department of Energy DOE	1631	1.13	Udice French Research Universities	597	1.04
Council of Scientific Industrial Research CSIR India	1331	0.93	Tongji University	534	0.93
Udice French Research Universities	1249	0.87	University of Chinese Academy of Sciences CAS	523	0.91
Tsinghua Univerisity	1206	0.84	Central South University	467	0.81
Consejo Superior de Investigaciones Cientificas CSIC	1142	0.79	Tsinghua Univerisity	448	0.78

**Table 5:** Top 10 scientific units that publish on the subject.

Waste Materials			Recycled Materials		
University/Institute	Number of Publications	[%]	University/Institute	Number of Publications	[%]
Concrete Science	10,179	15.500	Concrete Science	5892	21.730
Water Treatment	7382	11.241	Sustainability Science	3673	13.546
Sustainability Science	5519	8.404	Asphalt	1780	6.565
Bioengineering	3204	4.879	Polymer Science	1155	4.260
Energy & Fuels	2590	3.944	Electrochemistry	1151	4.245
Herbicides, Pesticides and Ground Poisoning	2312	3.520	Water Treatment	972	3.585
Paper and Wood Materials Science	2285	3.479	Photocatalysts	908	3.349
Soil Science	2140	3.259	Paper and Wood Materials Science	750	2.766
Electrochemistry	1924	2.930	Mineral and Metal Processing	741	2.733
Asphalt	1567	2.386	Energy and Fuels	579	2.135

## Data Availability

All data is available for review from the authors of the article.
